# Diagnostic value of genetic and epigenetic biomarker panels for colorectal cancer detection: a systematic review

**DOI:** 10.1007/s00384-025-04904-y

**Published:** 2025-05-22

**Authors:** Georgios Alampritis, Sarah Nohelia Thoukididou, Maria Ramos, Pantelis Georgiou, Melpomeni Kalofonou, Constantinos Simillis

**Affiliations:** 1 Department of Surgery, University of Cambridge, Cambridge, UK; 2 Department of Electrical and Electronic Engineering, Imperial College London, London, UK; 3 Cambridge Colorectal Unit, Addenbrookes Hospital, Cambridge University Hospitals NHS Foundation Trust, Cambridge, UK

**Keywords:** Colorectal cancer, Detection, Biomarker panels, Genetic, Epigenetic, CtDNA

## Abstract

**Purpose:**

Exploration of effective screening methods is imperative to improve current screening for colorectal cancer (CRC). Our aim was to systematically search the literature to identify and assess the diagnostic accuracy of both genetic and epigenetic biomarker panels for CRC detection using liquid biopsies for circulating tumour DNA (ctDNA) from stool, blood, or urine.

**Methods:**

A systematic review was performed according to the Preferred Reporting Items for Systematic Reviews and Meta-analyses (PRISMA) with searches in Medline, Embase, CENTRAL, and Web Of Science from inception up to March 20, 2025, using pre-defined keywords. Study quality assessment was performed using QUADAS-2 tool (Quality Assessment for Diagnostic Accuracy Studies 2). Primary and secondary outcomes were panel performance (sensitivity and specificity) for CRC, advanced precancerous lesions (APL), and staging of disease.

**Results:**

Forty-four studies were included. Exceptional performance for both CRC (sensitivity and specificity) and APL (sensitivity) was displayed by biomarker panels including methylated SDC2 with methylated SFRP1/2 (CRC: 91.5%/97.3%, APL: 89.2%) or methylated TFPI2 (CRC: 94.9%/98.1%, APL: 100%), and a 5-biomarker panel of mutational targets APC, Bat-26, KRAS, L-DNA, and p53 (CRC: 91.0%/93.0%, APL: 82.0%). Suboptimal APL sensitivities up to 57.0% were exhibited by Cologuard and variant panels (including KRAS, methylated BMP3, methylated NDRG4, FIT), and 47.8% for combinations including methylated SEPT9.

**Conclusions:**

High-performance, candidate ctDNA biomarker panels with exceptional diagnostic accuracy for both CRC and APL have been identified. Further work should focus on the development of large-scale studies to justify their clinical implementation.

## Introduction

### Colorectal cancer background

Improved understanding of the pathophysiology of colorectal cancer (CRC) is a promising avenue for efficient and fast-tracked screening. CRC ranks third in cancer incidence worldwide and is the second highest cancer-related cause of death, estimated at 935,000 deaths in 2020 [1]. These rates vary geographically with the highest seen in the most developed countries.

With the evolving field of evidence, CRC has been suggested to be a marker of socioeconomic development due to the trend of uniform rise in incidence rates with increasing Human Development Index (HDI) in countries undergoing major economic transition [2, 3]. Development of CRC is attributable to both hereditary and environmental risk factors. The upward incidence in transitioning countries reflects changes in modifiable environmental factors such as lifestyle and diet including increased level of cigarette smoking, intake of processed animal products including red meat, decreased physical activity, excess body fat, and heavy alcohol consumption [4].

A proportion of high-incidence countries have experienced a decline in colorectal cancer rates due to public health campaigns incorporating screening programmes and population-based education regarding healthier lifestyles. Increased colonoscopy screenings alongside stool-based haemoglobin detection, has supported the detection and removal of precursor lesions [5, 6]. Despite the promising trends for adults over 50 years old, it has been noticed that a higher proportion of adults under 50 years present with early-onset colorectal cancers with the incidence rising by 1–4% per year depending on the region [7, 8]. The rising incidence and burden of early-onset cancers (age < 50 years) has forced the American Cancer Society to lower the recommended screening age for average risk individuals from 50 to 45 years in 2018 [9].

CRC cancer prognosis and long-term survival is highly influenced by the TNM staging at time of diagnosis. Right-sided CRCs typically present with anaemia, fatigue, weight loss, and abdominal pain, or cramping, whilst left-sided CRCs with rectal bleeding and change in bowel habit [10, 11]. A substantial proportion of patients are asymptomatic and only 14% of CRCs are diagnosed at an early stage, highlighting the importance of effective screening and improving early diagnosis [12].

CRC is a heterogenous disease with several carcinogenic pathways exhibiting a variety of genetic and epigenetic alterations [13]. Three major precursor lesion pathways are recognised: Chromosomal Instability (CIN), Microsatellite Instability (MSI), and the Serrated Pathway/CpG Island Methylator Pathway (CIMP). The CIN pathway represents the traditional adenoma-carcinoma sequence model, primarily associated with left-sided CRCs, featuring activating mutations in proto-oncogenes such as KRAS (Kirsten rat sarcoma viral oncogene homolog) and inactivating mutations in tumour suppressor genes such as APC (adenomatous polyposis coli) and subsequently TP53 (tumour protein p53) [14, 15] In contrast, the MSI pathway is more prevalent in right-sided CRCs and is marked by defective DNA mismatch repair (MMR), leading to hypermutated tumours and genomic instability due to the accumulation of single nucleotide mutations [16, 17]. Lastly, the Serrated Pathway is characterised by hypermethylation of genes and BRAF (B-Raf proto-oncogene) mutations, resulting in enhanced MAPK/ERK (mitogen-activated protein kinase/extracellular signal-regulated kinase) signalling and uncontrolled proliferation, with further hypermethylation of the tumour suppressor gene p16 promoting carcinogenesis [18, 19]. Nevertheless, each tumour has its unique genomic profile, implying that there is interaction between the aforementioned pathways in the carcinogenesis route.

### Critical literature review

The current CRC screening methods include colon structure-based, image-based, and biological sample-based tests. The gold standard and current standard of care of CRC screening is colonoscopy, with high sensitivity and specificity, and potential for direct removal of early cancers and precancerous lesions at the time of detection. The disadvantages of colonoscopy include invasiveness, high costs, risk of bowel perforation, need for bowel preparation, and possible sedation which have direct impact on the uptake of the test and participation in screening programmes [20]. The effectiveness of a screening program depends not only on the screening test performance, but also on patient adherence to achieve a high participation rate, which is affected by the burden of the test, risk of complications, the cost, cultural beliefs of the individual and logistics of carrying out the test [6]. CT Colonography can be a useful semi-invasive alternative in selected cases; however, it has its own disadvantages, including ionising radiation, full bowel preparation, high costs, dependence on radiologist’s technical expertise for accurate interpretation, and the requirement for follow-up colonoscopy in the event of abnormalities. Consequently, it is not considered appropriate for population screening [21].

Non-invasive stool tests detecting faecal haemoglobin, the guaiac faecal occult blood test (gFOBT) and the faecal immunochemical test (FIT), are alternatives currently being used in two-step screening programmes. The FIT is the preferred method of screening over gFOBT due to its superior test characteristics (higher sensitivity, one stool sample needed for multiple FIT tests), lack of need for medication or dietary changes, and greater adherence rates [22, 23]. Additionally, a meta-analysis reported a pooled FIT sensitivity of 79% and specificity of 94%, with however large heterogeneity in included studies due to the use of different cut-offs for a positive result [23]. Despite a demonstrated overall reduction in CRC mortality by FOBT-based organised screening programmes, the FOBTs (FIT and gFOBT) display a relatively low performance for detecting precancerous lesions and thus are mainly used for detecting advanced colorectal neoplasms [24].

Recent advancements in CRC detection strategies utilise liquid biopsies for high yield, non-invasive tests. Liquid biopsies consist of a sample of any bodily fluid that contain genetic material from a tumour such as blood, faeces, or urine. Compared to single tissue biopsy, they provide a better characterisation of cancer genome [25]. They use circulating materials such as circulating tumour DNA (ctDNA; cell-free DNA of tumour origin), circulating tumour cells (CTCs), cell-free miRNA, and cell-derived vesicles (such as exosomes) to detect molecular alterations present in carcinogenesis [25–27]. ctDNA compared to CTCs in cancer patients, was observed to be more specific for DNA mutations and in higher numbers [28]. Specifically, ctDNA is derived from apoptotic and necrotic tumour cells releasing fragmented DNA into the circulation or stool, reflecting the genetic and epigenetic alterations of the original tumour cells and their corresponding microenvironment [26, 27, 29]. Interestingly, in some instances, the early stages of carcinogenesis are marked by many epigenetic changes before any somatic mutations and histopathological changes can be detected [30]. Hence, the combinatory mutational and epigenetic analysis of liquid biopsies is representing a promising tool with mass screening potential.

Cologuard® is the first FDA-approved multitarget stool DNA test in 2014 (mt-sDNA) that combines both DNA and FIT testing for detection of abnormal DNA and faecal haemoglobin in stool samples. The DNA molecular assays test for methylated BMP3 and NDRG4, mutant KRAS and β-actin (reference gene). In a large multi-centre, cross-sectional study reported by Imperiale et al. [31]*,* the higher sensitivity for CRC (92.3% vs 73.8%) and advanced adenomas (42.4% vs 23.8%) is accompanied by a lower specificity (86.6% vs 94.9%), compared to FIT testing alone. Apart from the higher costs when using multi-target panels, a higher number of patients were excluded due to problematic faecal DNA testing (*n* = 689; sample integrity, technical failure, missing samples) compared to FIT testing alone (*n* = 34; insufficient haemoglobin sample) which highlights the complexity of stool sample collection and analysis. This further highlights the necessity of investigating sources beyond stool for ctDNA panels.

### Rational, aim, and objectives

The use of individual biomarkers has been shown to yield an inferior performance compared to the combination of several biomarkers in a panel. Since the approval of Cologuard®, several other candidate biomarkers panels (including variations of mt-sDNA) using liquid biopsies have been extensively investigated in an attempt to identify a superior panel comprised of both genetic and epigenetic biomarkers. Special consideration for those panels should be a higher performance for the detection of advanced precancerous lesions, including advanced adenomas, as this can benefit early detection and cure. To the best of our knowledge, the current literature lacks a systematic reporting of liquid biopsy biomarker panels that extend beyond a single ctDNA source (whether from faecal or blood material) or a specific focus on DNA methylation. Thus, our aim was to systematically search the literature to identify and assess the diagnostic accuracy, in terms of sensitivity and specificity, of genetic and epigenetic ctDNA biomarkers panels for CRC detection using specimens of stool, blood, or urine. Additionally, to further determine the extent of detecting advanced precancerous lesions and differentiating between different stages of CRC disease (I–IV).

## Methodology

### Search strategy

A systematic literature review was conducted in accordance with the Preferred Reporting Items for Systematic Reviews and Meta-analyses (PRISMA) guidelines and was registered in PROSPERO (CRD420251017969), where the review protocol can be accessed [32]. Systematic searches were performed in four main bibliographic databases: Medline, Embase, CENTRAL, and Web Of Science from inception up to March 20, 2025. The search query included the following keywords starting with five isolated searches and lastly combining the searches for each database: “(colorectal cancer OR colon cancer OR rectal cancer) AND (biomarker OR genetic OR epigenetic OR methylated DNA OR hypermethylated DNA) AND (circulating tumour DNA OR plasma cell-free DNA OR liquid biopsy OR stool DNA) AND (screening OR diagnosis OR detection OR prediction OR prognosis OR treatment OR management) AND (panel* OR multi-gene OR multigene OR multi-target OR multitarget OR multi-test OR multitest)”. All articles published in the English language in peer-review academic journals were selected for further review. Additionally, references of systematic reviews and meta-analyses were searched manually for identification of any other eligible studies.

### Eligibility assessment

Screening of articles was performed by two independent reviewers (GA, ST) through titles, abstracts, and full-texts against the pre-defined eligibility criteria. Any conflicts were resolved by a third reviewer. Studies were included if they reported on the performance (sensitivity and specificity) of a ctDNA biomarker panel (defined as having at least 2 biomarkers – genetic and/or epigenetic) for detecting CRC (and also advanced precancerous lesions if applicable).

Our exclusion criteria were as follows: (1) studies on any type of cancer other than colorectal, (2) literature reviews, systematic reviews, meta-analyses, conference abstracts, editorials, doctoral theses, letters to the editor and comments, (3) studies not focusing on genetic (mutation) and epigenetic (methylation) biomarker panels for screening, detection, or diagnosis of colorectal cancer; also, those reporting only for individual biomarkers, (4) wrong outcome such as treatment response/monitoring, prognosis, cost-effectiveness, post-treatment recurrence, pre/post-treatment phase, (5) any sources of DNA other than blood, stool, or urine; also, no source of DNA reported, using non-ctDNA from blood samples (e.g., cf-nucleosomes, miRNAs) and proteomics, (6) studies without human samples including in silico studies, animal models, or cell-lines, (7) data only for advanced precancerous lesions, (8) studies with inflammatory bowel disease or hereditary CRC syndrome patients, and (9) studies that did not report sample sizes.

### Data extraction and analysis

Primary outcomes of interest were composite sensitivity and specificity of biomarker panels for CRC detection with sensitivity defined as the percentage of CRC patients with detected ctDNA aberrations and specificity defined as the percentage of healthy individuals without detected ctDNA aberrations. Secondary outcomes included performance to detect advanced precancerous lesions (APLs) defined as advanced adenomas of ≥ 1 cm diameter or villous architecture on histology or high-grade dysplasia, and serrated polyps ≥ 1 cm, and CRC at different stages of disease determined by the AJCC (American Joint Committee on Cancer) staging system [33, 34]. Additionally, data were extracted for patient characteristics (sample size, age, sex), ctDNA source, method of analysis, biomarkers included and study type (case–control or cohort type of cross-sectional study) using a validated algorithm [35], for diagnostic accuracy studies. Data were tabulated in Excel Version 16.85.

### Quality assessment

Quality assessment of all included studies was performed using the validated Quality Assessment for Diagnostic Accuracy Studies 2 tool (QUADAS-2) to evaluate risk of bias and applicability concerns for four key domains: patient selection, index tests, reference standards, and flow and timing. Signalling questions were used to assist in rating the domains as “low”, “high”, or “unclear” in terms of risk of bias and applicability. Review Manager 5 software Version 5.4 was used for managing the QUADAS-2 data and creating figures [36].

## Results

### PRISMA flow diagram

**Fig. 1:**
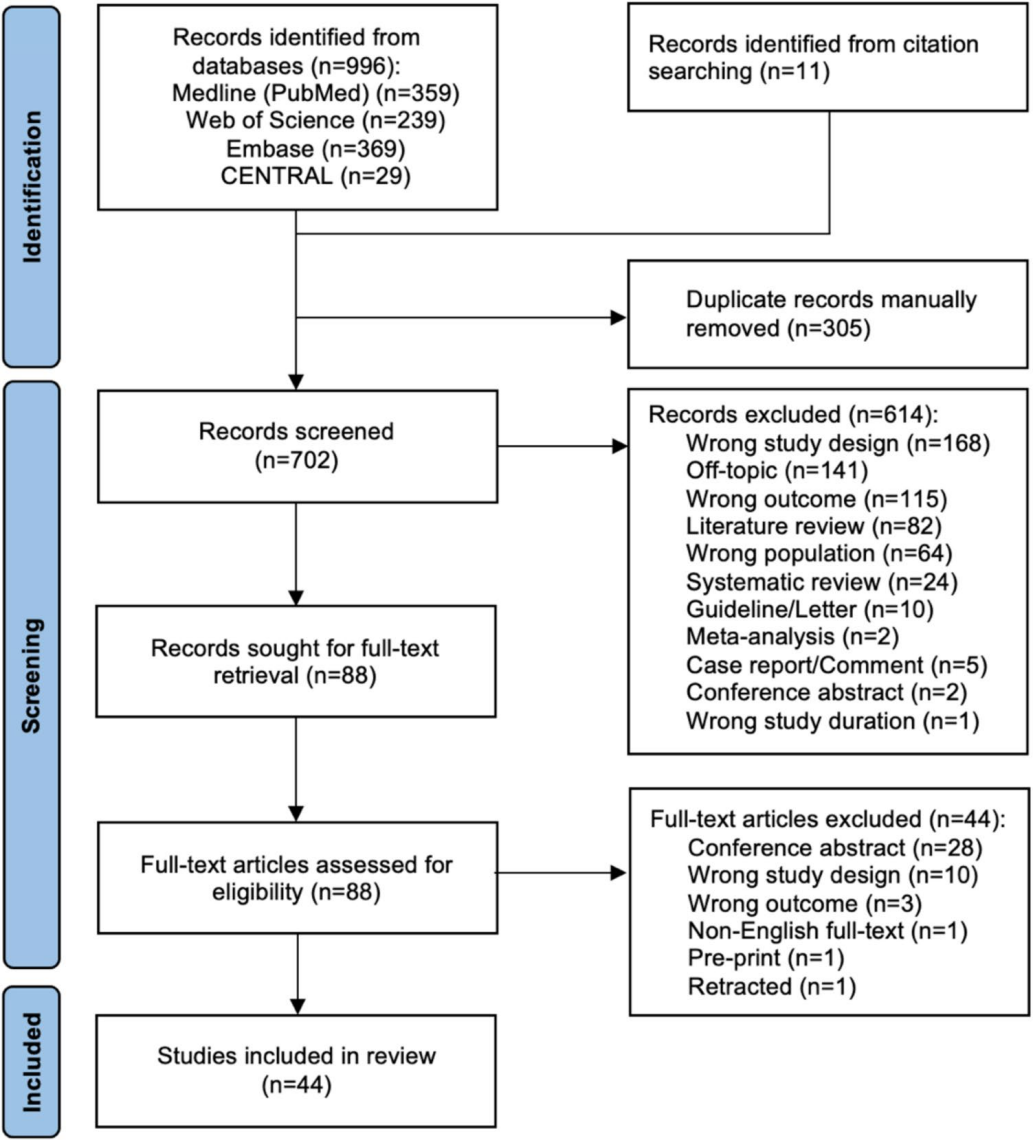
The Preferred Reporting Items for Systematic Reviews and Meta-Analyses (PRISMA) flow diagram for the inclusion of the studies

The systematic search yielded a total of 1007 eligible abstracts, with 996 from four databases and 11 identified through citation searching. After duplicate removal (*n* = 305), 702 articles were screened by title and abstract for eligibility, excluding 614 for various reasons leaving 88 articles for full-text retrieval (Fig. 1). From those, 44 further articles were eliminated resulting to 44 articles to be included in the review, based on our inclusion and exclusion criteria.

### Quality assessment of included studies

**Fig. 2:**
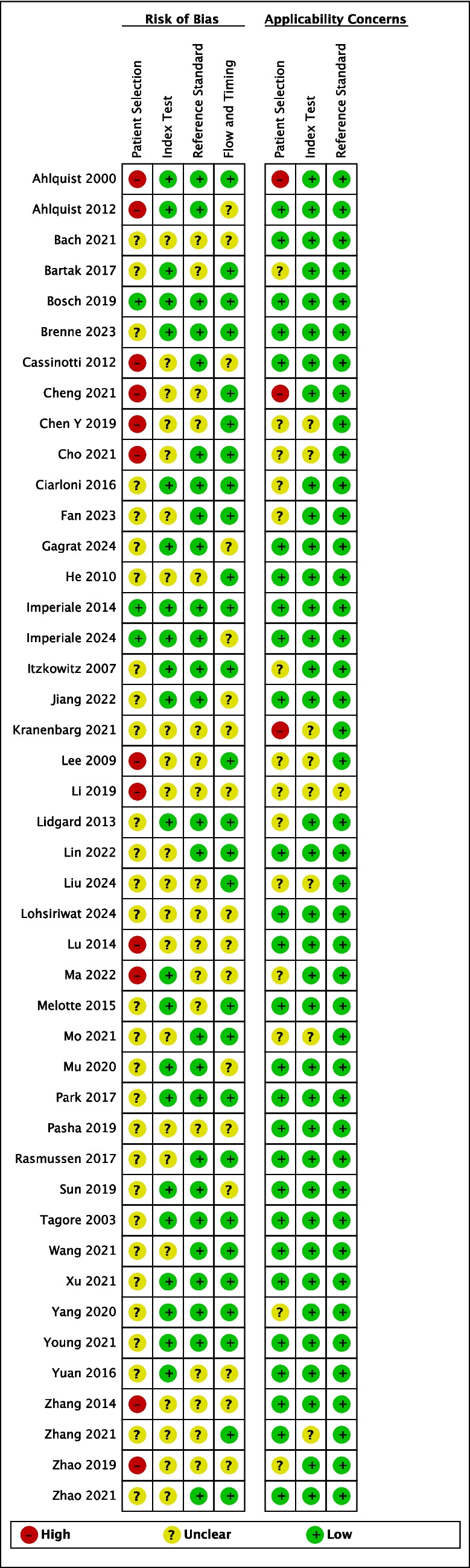
Risk of bias and applicability concerns summary: review authors’ judgements about each domain for each included study

**Fig. 3:**
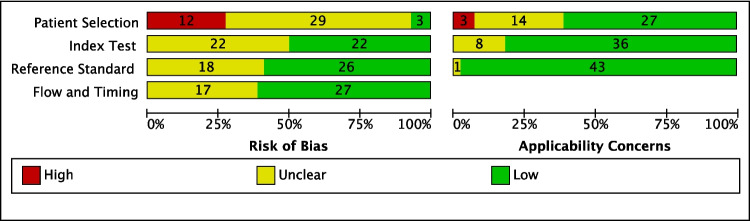
Risk of bias and applicability concerns graph: review authors’ judgements about each domain presented as percentages and counts across included studies

Only 2 studies scored low risk in all domains of the quality assessment on the QUADAS-2 tool (Fig. 2) [31, 37]*.* The majority of studies (*n* = 42) scored unclear or high risk of bias on at least one domain, predominantly patient selection with 66% (*n* = 29) unclear and 27% (*n* = 12) high risk of bias (Fig. 3). This was due to several case–control study designs and lack of detailed information on the nature of patient selection process, especially on the sampling method employed, which may introduce sampling bias. In the other 3 domains of risk of bias (index test, reference standard, and flow and timing), more than half of the papers scored low risk of bias. An unclear rating was due to incomplete information on the use of a pre-specified panel cut-off value, colonoscopy process and timing, or blinding during interpretation of index and reference standard. In terms of applicability concerns, many papers (59%, *n* = 26) scored low risk in all domains, with several papers lacking again (high or unclear risk) on the patient selection domain (39%, *n* = 17) due to the patient selection process and study design.

### Study characteristics

The systematic review includes 44 studies with a total sample size of 30803 consisting of 48% (14047) male and 52% (14936) female participants (Table 1). The weighted mean (± SD) age of participants amongst all the studies was 61 ± 5 years. The mean age amongst specific groups, was 64 ± 4 years for the CRC group, 63 ± 4 years for the APL group, and 58 ± 9 years for the control group.

**Table 1:** Characteristics of included studies

Study	Cross-sectional study subdesign	Biomarkers		ctDNA source	n	Sex (M/F)	Age			
							μ	CRC	APL	C
Ahlquist 2000 [38]	Case–control type	APC, Bat-26, KRAS, L-DNA, p53		Stool	61	30/31	70	70	73	68
Ahlquist 2012 [39]	Case–control type	KRAS, mBMP3, mNDRG4, mTFPI2, mVIM, β-actin, Haemoglobin		Stool	678	339/339	60	-	-	57
Bach 2021 [40]	Case–control type	mSDC2, mSEPT9		Urine	155	76/79	65	66	-	62
Bartak 2017 [41]	Case–control type	mPRIMA1, mSDC2, mSFRP1, mSFRP2		Plasma	121	-	-	-	-	-
Bosch 2019 [37]	Cohort type	KRAS, mBMP3, mNDRG4, β-actin, Haemoglobin/FIT (MT-sDNA)		Stool	1014	520/494	60	-	-	-
Brenne 2023 [42]	Case–control type	mBMP3, mFLI1, mIKZF1, mNPTX2, mSFRP1, mSFRP2, mSLC8 A1, mVIM		Plasma	143	66/77	70	70	-	70
Cassinotti 2012 [43]	Case–control type	mCYCD2, mHIC1, mPAX5, mRASSF1 A, mRB1, mSRBC		Plasma	90	-	64	68	62	61
Chen Y 2019 [44]	Case–control type	mSDC2, mSEPT9 (ColoDefense)		Serum	225	-	47	61	-	33
Cheng 2021 [45]	Case–control type	mADHFE1, mPPP2R5 C, mSDC2		Stool	30	21/9	55	63	63	41
Cho 2021 [46]	Case–control type	mFAM123 A, mGLI3, mPPP1R16B, mSLIT3, mTMEM90B		Plasma	157	-	-	-	-	-
Ciarloni 2016 [47]	Case–control type	29 gene panel, CEA, CYFRA21-1		Plasma	349	194/155	65	69	67	61
Fan 2023 [48]	Case–control type	KRAS, mBMP3, mNDRG4, β-actin, Haemoglobin/FIT (ColoClear MT-sDNA), CEA, 6 genera of gut microbiota		Stool	105	61/44	60	65	-	54
Gagrat 2024 [49]	Case–control type	mLASS4, mLRRC4, mPPP2R5 C, mZDHHC1, Haemoglobin		Stool	777	387/390	64	-	-	63
He 2010 [50]	Case–control type	mALX4, mSEPT9, mTMEFF2		Plasma	352	237/115	59	58	-	60
Imperiale 2014 [31]	Cohort type	KRAS, mBMP3, mNDRG4, β-actin, Haemoglobin/FIT (Cologuard MT-sDNA)		Stool	9989	4625/5364	64	-	-	-
Imperiale 2024 [51]	Cohort type	mLASS4, mLRRC4, mPPP2R5 C, mZDHHC1, Haemoglobin		Stool	7662	3402/4260	64	-	-	-
Itzkowitz 2007 [52]	Case–control type	mVIM, DIA (DNA Integrity Assay)		Stool	162	86/76	60	66	-	59
Jiang 2022 [53]	Case–control type	mPAX8, mRASSF1, mSFRP2, Haemoglobin/FIT		Stool	250	129/121	69	69	-	68
Kranenbarg 2021 [54]	Case–control type	mAKR1B1, mCOL6 A2, mMAL, mTMEFF2, mZNF671		Plasma	40	24/16	63	56	-	70
Lee 2009 [55]	Case–control type	mAPC, mMGMT, mRASSF2 A, mWIF1		Plasma	583	-	-	61	-	58
Li 2019 [56]	Case–control type	80 marker panel (including mSEPT9, mIKZF1)		Plasma	283	150/133	62	65	-	60
Lidgard 2013 [57]	Case–control type	KRAS, mBMP3, mNDRG4, β-actin, Haemoglobin/FIT (MT-sDNA)		Stool	1003	454/549	65	-	-	65
Lin 2022 [58]	Case–control type	APC, BRAF, KRAS, mSDC2, mSFRP2		Stool	292	164/128	58	-	-	-
Liu 2024 [59]	Case–control type	mSDC2, mSEPT9, mVIM		Stool	33	-	-	-	-	-
Lohsiriwat 2024 [60]	Cohort type	mADHFE1, mSDC2, mPPP2R5 C		Stool	274	108/166	62	-	-	-
Lu 2014 [61]	Case–control type	mGATA4/5, mNDRG4, mSFRP2, mVIM		Stool	96	45/51	60	60	-	60
Ma 2022 [62]	Case–control type	mNDRG4, mSDC2, mTFPI2, mWIF1 (ColoCaller)		Stool	158	-	-	-	-	-
Melotte 2015 [63]	Unclear	mFOXE1, mSYNE1		Plasma	306	-	-	-	-	-
Mo 2021 [64]	Case–control type	BRAF, KRAS, PI3 KCA, mBMP3, mNDRG4, mSEPT9, FIT, Fusobacterium nucleatum, Parvimonas micra		Stool	162	94/68	58	-	-	-
Mu 2020 [65]	Case–control type	KRAS, mBMP3, mNDRG4, β-actin, Haemoglobin/FIT (ColoClear MT-sDNA)		Stool	839	445/394	59	62	60	57
Park 2017 [66]	Case–control type	mBMP3, mNDRG4, mSFRP2, mTFPI2		Stool	111	71/38	60	61	63	56
Pasha 2019 [67]	Case–control type	mRUNX3, mSFRP1, CEA		Stool	165	106/59	-	-	-	-
Rasmussen 2017 [68]	Case–control type	mALX4, mBMP3, mNPTX2, mRARB, mSDC2, mSEPT9, mVIM, female, age > 66		Plasma	295	174/121	67	68	-	65
Sun 2019 [69]	Case–control type	KRAS, mSDC2, mSFRP2, Haemoglobin		Stool	233	124/109	-	-	-	-
Tagore 2003 [70]	Cohort type	APC, BAT-26, KRAS, p53, DIA (DNA Integrity Assay)		Stool	292	138/154	63	64	61	62
Wang 2021 [71]	Unclear	mBMP3, mNDRG4, mSDC2, Haemoglobin (sDNA-FOBT)		Stool	144	83/61	58	63	-	55
Xu 2021 [72]	Cohort type	mBCAT1, mSDC2, mSEPT9, CEA, Haemoglobin/FIT		Plasma, Stool	294	192/102	61	65	-	60
Yang 2020 [73]	Case–control type	KRAS, mNDRG4, mSDC2, mTFPI2, β-actin		Stool	151	71/80	61	60	59	65
Young 2021 [74]	Unclear	mBCAT1, mIKZF1, mIRF4		Plasma	1620	902/718	63	67	-	60
Yuan 2016 [75]	Case–control type	mOSMR, mSEPT9		Plasma	321	163/158	62	64	-	61
Zhang 2014 [76]	Case–control type	mSFRP2, mWIF1		Stool	145	-	-	-	-	-
Zhang 2021 [77]	Case–control type	mSDC2, mTFPI2		Stool	130	76/54	55	59	-	49
Zhao 2019 [78]	Case–control type	mSDC2, mSEPT9 (ColoDefense)		Plasma	384	214/170	50	62	59	37
Zhao 2021 [79]	Case–control type	mSDC2, mSFRP2 (SpecColon)		Stool	129	76/53	60	61	67	44
			***Total/Mean (if applicable): 30,803 14,047/14936***				***61***	***64***	***63***	***58***
			***Standard Deviation:***				***5***	***4***	***4***	***9***

*n*, sample size; *M/F*, males/females; *μ*, weighted mean of participants; *CRC*, colorectal cancer; *APL*, advanced adenomas of ≥ 1 cm diameter or villous architecture on histology or high-grade dysplasia; and serrated polyps ≥ 1 cm; *C*, controls; *‘’- ‘’*, not specified/cannot be inferred from data. The pooled comparisons were performed using the available data from full texts or supplementary materials, but despite this there were instances data were not reported or have not been specific enough to be included in the inter-group comparisons

There were 35 (80%) case–control type cross-sectional studies, 6 (14%) cohort-type cross-sectional studies and 3 (6%) studies with an unclear design. Few studies stated the exact study design in their full-text; for these we had to derive the cross-sectional sub design using the available information in the methodology section. In some cases, methodological information including chronological details was limited.

**Fig. 4:**
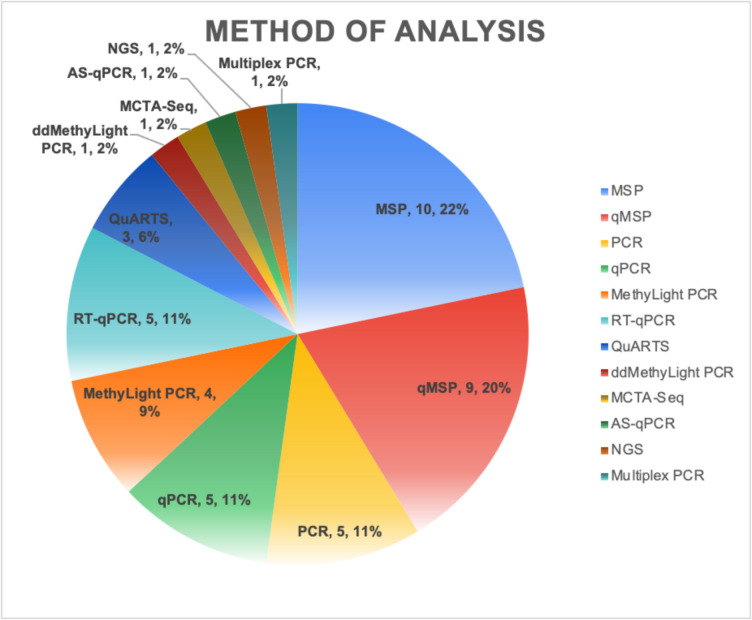
Count and percentage of different ctDNA analysis methods amongst studies. ctDNA = circulating tumour DNA, PCR = polymerase chain reaction, qPCR = quantitative PCR, MSP = methylation specific PCR, qMSP = quantitative methylation specific PCR, ddMethyLight PCR = droplet digital MethyLight PCR, MCTA-Seq = methylated CpG tandem amplification and sequencing, AS-qPCR = allele specific-quantitative PCR, NGS = next-generation sequencing, QuARTS = quantitative allele-specific real-time target and signal amplification, RT-qPCR = reverse transcription-quantitative PCR

With regards to the circulating tumour DNA source (ctDNA), there were 27 (62%) studies using stool samples only, 15 (34%) studies using blood samples only (14 plasma and 1 serum), 1 (2%) study with urine sample, and 1 (2%) study with both plasma and stool samples. There were several methods used to analyse ctDNA, the majority of which were PCR-based (Fig. 4, Table 2). The most commonly used were MSP (Methylation Specific Polymerase Chain Reaction) in 22% (*n* = 10) and quantitative MSP in 20% (*n* = 9), followed by PCR and quantitative PCR in 11% (*n* = 5), respectively for each. The remaining eight methods occupy 36% (*n* = 17).

**Table 2:** Performance (sensitivity = sens & specificity = spec) of reported biomarker panels for colorectal cancer (CRC) and advanced precancerous lesions (APL)

Study	Biomarkers	ctDNA source	Method	CRC panel		Stage sensitivity, %				APL sensitivity,% (n)
				Sens, % (n)	Spec, % (n)	I	II	III	IV	
Ahlquist 2000 [38]	APC, Bat-26, KRAS, L-DNA, p53	Stool	PCR	91.0 (20/22)	93.0 (26/28)	-	-	-	-	82.0 (9/11)
Ahlquist 2012 [39]	KRAS, mBMP3, mNDRG4, mTFPI2, mVIM, β-actin, Haemoglobin	Stool	QuARTS	85.0 (214/252	89.0 (261/293)	84.0	80.0	95.0	69.0	54.0 (72/133)
Bach 2021 [40]	mSDC2, mSEPT9	Urine	qMSP	70.0 (64/92)	86.0 (54/63)	-	-	-	-	-
Bartak 2017 [41]	mPRIMA1, mSDC2, mSFRP1, mSFRP2	Plasma	MethyLight PCR	91.5 (43/47)	97.3 (36/37)	-	-	-	-	89.2 (33/37)
Bosch 2019 [37]	KRAS, mBMP3, mNDRG4, β-actin, Haemoglobin/FIT (MT-sDNA)	Stool	PCR	85.7 (6/7)	89.1 (791/888)	-	-	-	-	47.8 (44/92)
Brenne 2023 [42]	mBMP3, mFLI1, mIKZF1, mNPTX2, mSFRP1, mSFRP2, mSLC8 A1, mVIM	Plasma	MSP	43.0 (31/72)	86.0 (61/71)	-	-	-	-	-
Cassinotti 2012 [43]	mCYCD2, mHIC1, mPAX5, mRASSF1 A, mRB1, mSRBC	Plasma	PCR	83.7 (−/30)	67.9 (−/30)	-	-	-	-	− [54.6 (−/30)^a^]
Chen Y 2019 [44]	mSDC2, mSEPT9 (ColoDefense)	Serum	qPCR	86.5 (96/111)	92.1 (105/114)	69.2	85.7	89.7	100.0	-
Cheng 2021 [45]	mADHFE1, mPPP2R5 C, mSDC2	Stool	qMSP	84.6 (11/13)	92.3 (12/13)	-	-	-	-	− [75.0 (3/4)^a^]
Cho 2021 [46]	mFAM123 A, mGLI3, mPPP1R16B, mSLIT3, mTMEM90B	Plasma	ddMethyLight PCR	57.3 (56/97)	95.0 (57/60)	35.3	54.2	45.5	95.7	-
Ciarloni 2016 [47]	29 gene panel, CEA, CYFRA21-1	Plasma	RT-qPCR	78.1 (57/73)	92.2 (83/90)	60.6		92.5		52.3 (34/65)
Fan 2023 [48]	KRAS, mBMP3, mNDRG4, β-actin, Haemoglobin/FIT (ColoClear MT-sDNA), CEA, 6 genera of gut microbiota	Stool	RT-qPCR	98.1 (−/54)	92.3 (−/51)	-	-	-	-	-
Gagrat 2024 [49]	mLASS4, mLRRC4, mPPP2R5 C, mZDHHC1, Haemoglobin	Stool	QuARTS	95.2 (−/112)	89.8 (−/176)	93.9	94.7	100.0	100.0	57.2 (−/98)
He 2010 [50]	mALX4, mSEPT9, mTMEFF2	Plasma	MethyLight PCR	81.0 (147/182)	90.0 (153/170)	-	-	-	-	-
Imperiale 2014 [31]	KRAS, mBMP3, mNDRG4, β-actin, Haemoglobin/FIT (Cologuard MT-sDNA)	Stool	PCR	92.3 (60/65)	89.8 (4002/4457)	93.3			-	42.4 (321/757)
Imperiale 2024 [51]	mLASS4, mLRRC4, mPPP2R5 C, mZDHHC1, Haemoglobin	Stool	RT-qPCR	93.0 (53/57)	88.5 (6214/7022)	92.0	100.0	83.3	75.0	48.4 (282/583)
Itzkowitz 2007 [52]	mVIM, DIA (DNA Integrity Assay)	Stool	MSP	87.5 (35/40)	82.0 (100/122)	75.0	90.0	94.1	80.0	-
Jiang 2022 [53]	mPAX8, mRASSF1, mSFRP2, Haemoglobin/FIT	Stool	MSP	80.0 (100/125)	93.6 (117/125)	73.2			88.9	-
Kranenbarg 2021 [54]	mAKR1B1, mCOL6 A2, mMAL, mTMEFF2, mZNF671	Plasma	MSP	-	100 (20/20)	-	-	-	100.0	-
Lee 2009 [55]	mAPC, mMGMT, mRASSF2 A, mWIF1	Plasma	MSP	86.5 (−/243)	92.1 (−/276)	-	-	-	-	− [74.6 (−/64)^a^]
Li 2019 [56]	80 marker panel (including mSEPT9, mIKZF1)	Plasma	MCTA-Seq	77.0 (−/147)	90.0 (−/136)	65.0	76.0	81.0		-
Lidgard 2013 [57]	KRAS, mBMP3, mNDRG4, β-actin, Haemoglobin/FIT (MT-sDNA)	Stool	QuARTS	98.0 (91/93)	90.0 (716/796)	95.0	100.0	97.0	100.0	57.0 (65/114)
Lin 2022 [58]	APC, BRAF, KRAS, mSDC2, mSFRP2	Stool	qMSP	88.6 (93/105)	88.4 (84/95)	88.9	83.3	88.5	100.0	75.0 (69/92)
Liu 2024 [59]	mSDC2, mSEPT9, mVIM	Stool	Multiplex PCR	91.4 (−/14)	100.0 (19/19)	100.0	91.3	-	-	-
Lohsiriwat 2024 [60]	mADHFE1, mSDC2, mPPP2R5 C	Stool	qMSP	91.5 (43/47)	90.3 (205/227)	-	-	-	-	29.4 (5/17)
Lu 2014 [61]	mGATA4/5, mNDRG4, mSFRP2, mVIM	Stool	MSP	96.4 (54/56)	65.0 (26/40)	-	-	-	-	-
Ma 2022 [62]	mNDRG4, mSDC2, mTFPI2, mWIF1 (ColoCaller)	Stool	qMSP	94.9 (37/39)	98.1 (105/107)	88.9	93.8	100.0	100.0	100.0 (6/6)
Melotte 2015 [63]	mFOXE1, mSYNE1	Plasma	qMSP	58.0 (38/66)	91.0 (219/240)	37.0	87.0	55.0	100.0	-
Mo 2021 [64]	BRAF, KRAS, PI3 KCA, mBMP3, mNDRG4, mSEPT9, FIT, Fusobacterium nucleatum, Parvimonas micra	Stool	NGS, qPCR	81.5 (88/108)	94.4 (34/36)	60.0	84.6	91.9	75.0	- [27.8 (5/18)^a^]
Mu 2020 [65]	KRAS, mBMP3, mNDRG4, β-actin, Haemoglobin/FIT (ColoClear MT-sDNA)	Stool	RT-qPCR	97.5 (198/203)	89.1 (384/431)	100.0	96.5	100.0	94.1	53.1 (26/49)
Park 2017 [66]	mBMP3, mNDRG4, mSFRP2, mTFPI2	Stool	MSP	94.3 (33/35)	55.0 (22/40)	88.2		100.0		72.2 (26/36)
Pasha 2019 [67]	mRUNX3, mSFRP1, CEA	Stool	MSP	84.7 (72/85)	67.5 (27/40)	-	-	-	-	-
Rasmussen 2017 [68]	mALX4, mBMP3, mNPTX2, mRARB, mSDC2, mSEPT9, mVIM, female, age > 66	Plasma	MSP	90.7 (175/193)	72.5 (74/102)	88.7		-	-	-
Sun 2019 [69]	KRAS, mSDC2, mSFRP2, Haemoglobin	Stool	MethyLight PCR, AS-qPCR	91.4 (96/105)	86.1 (93/108)	87.5	100.0	-	-	60.0 (12/20)
Tagore 2003 [70]	APC, BAT-26, KRAS, p53, DIA (DNA Integrity Assay)	Stool	PCR	63.5 (33/52)	96.2 (204/212)	75.0	66.7	41.7	50.0	57.1 (16/28)
Wang 2021 [71]	mBMP3, mNDRG4, mSDC2, Haemoglobin (sDNA-FOBT)	Stool	qPCR	85.4 (41/48)	92 (46/50)	71.4	88.9	80.0	100.0	− [85.7 (6/7)^a^]
Xu 2021 [72]	mBCAT1, mSDC2, mSEPT9, CEA, Haemoglobin/FIT	Plasma, Stool	RT-qPCR	84.6 (−/104)	95.4 (−/−)	-	-	-	-	-
Yang 2020 [73]	KRAS, mNDRG4, mSDC2, mTFPI2, β-actin	Stool	qMSP	90.0 (45/50)	94.0 (47/50)	91.9			84.6	70.6 (36/51)
Young 2021 [74]	mBCAT1, mIKZF1, mIRF4	Plasma	qPCR	73.9 (136/184)	90.1 (739/820)	39.0	87.7	78.4	84.8	15.7 (53/337)
Yuan 2016 [75]	mOSMR, mSEPT9	Plasma	MethyLight PCR	77.0 (144/187)	81.7 (89/109)	78.1		76.8		− [28.0 (7/25)^p^]
Zhang 2014 [76]	mSFRP2, mWIF1	Stool	MSP	81.3 (39/48)	96.7 (29/30)	85.7	80.0	71.4	100.0	80.0 (12/15)
Zhang 2021 [77]	mSDC2, mTFPI2	Stool	qMSP	93.4 (57/61)	94.3 (50/53)	92.3		94.3		− [81.3 (13/16)^a^]
Zhao 2019 [78]	mSDC2, mSEPT9 (ColoDefense)	Plasma	qMSP	88.9 (104/117)	92.8 (154/166)	80.0	90.0	89.5	100.0	47.8 (11/23)
Zhao 2021 [79]	mSDC2, mSFRP2 (SpecColon)	Stool	qPCR	89.7 (52/58)	89.5 (34/38)	88.9	93.3	83.3	100.0	61.5 (8/13)

*ctDNA source*, circulating tumour DNA source; *PCR*, polymerase chain reaction; *qPCR*, quantitative PCR; *MSP*, methylation specific PCR; *qMSP*, quantitative methylation specific PCR; *ddMethyLight PCR*, droplet digital MethyLight PCR; *MCTA-Seq*, methylated CpG tandem amplification and sequencing; *AS-qPCR*, allele specific-quantitative PCR; *NGS*, next-generation sequencing; *QuARTS*, quantitative allele-specific real-time target and signal amplification; *RT-qPCR*, reverse transcription-quantitative PCR; *a*, adenoma data; *p*, polyp data; ‘’- ‘’, not specified/cannot be inferred from data; *n*, sample; *m*, methylated

### Panel performance for colorectal cancer and advanced precancerous lesions

From the 44 studies, the CRC overall sensitivities ranged from 43.0 to 98.1% and the specificities from 55.0 to 100%. With regards to the APL, sensitivities amongst studies ranged from 15.7 to 100%. Furthermore, the majority of CRC stage sensitivities (I**–**IV) for each study showed an increase with higher disease stages. The sensitivities ranged from 35.3 to 100% for stage I, 54.2**–**100% for stage II, 41.7**–**100% for stage III, and 50**–**100% for stage IV. The biomarker panel performance of each individual study is summarised in Table 2, along with the respective outcome measures for the panel’s accuracy for CRC and APL detection.

#### KRAS, mBMP3, mNDRG4, and haemoglobin panels (7 studies)

Several reported studies using variants of the Cologuard® stool test in an attempt to replicate or improve the biomarker panel. Six studies utilised the 4-biomarker strategy of KRAS, methylated BMP3, methylated NDRG4, and faecal haemoglobin (including β-actin as reference gene) with stool samples, showing overall CRC sensitivities of 81.5–98.1% with specificities of 89.0–94.4% [31, 37, 39, 48, 57, 64, 65]. The biomarker panels detected APLs with moderate performance (42.4–57.0%). The most promising biomarker combination from this cohort remains the classic 4-biomarker panel with the highest performance achieved by Lidgard et al. (case–control design) [57], with CRC sensitivity and specificity of 98% (91/93) and 90% (716/796), respectively, and an APL sensitivity of 57% (65/114). For Stage I to IV CRC, sensitivities were 95%, 100%, 97%, and 100%. Attempts to improve the current 4-biomarker panel by adding further methylated genes by Alquist et al. [39] and even stool bacteria level detection (microbial dysbiosis) by Mo et al. [64], proved to be unsuccessful in increasing the overall performance of the panel in those studies. Despite, this Fan et al. combined the 4-biomarker panel with CEA and 6 genera of gut microbiota, enhancing performance to 98.1% (−/53) sensitivity and 92.3% (−/51) specificity. However, their findings did not include APLs [48].

#### mSDC2 and mSEPT9 panels (6 studies)

The 2-biomarker panel of methylated SDC2 and methylated SEPT9, being another common strategy, was used in isolation by three studies [40, 44, 78], while three other studies combined it with additional mutational and/or epigenetic markers [59, 68, 72]. The panels achieved overall CRC sensitivities between 70.0–91.4% and specificities of 72.5–100%, with only Zhao et al. [78] reporting an APL sensitivity (47.8%). The 2-biomarker panel of mSDC2 and mSEPT9 is referred commercially as ColoDefense®, and Zhao et al.’s study demonstrated the best overall performance out of the cohort with 88.9% (104/117) and 92.8% (154/166) CRC sensitivity and specificity, and a moderate performance of 47.8% (11/23) for APL. For Stage I to IV CRC, sensitivities were 80%, 90%, 89.5%, and 100%. The three studies combining the 2-biomarker panel with other markers [59, 68, 72], reported sensitivities of 91.4%, 90.7%, and 84.6%, and specificities of 100%, 72.5%, and 95.4%, respectively, without evaluating accuracy for APL detection. While some combinations improved performance, the variability in findings suggests that, at present, the classic 2-biomarker mSDC2 and mSEPT9 panel remains the most reliable choice for overall CRC detection in the group, particularly as it is the only one to have reported results for APL detection.

#### mSDC2 and mSFRP1/2 panels (4 studies)

High performance was exhibited by a biomarker panel combination including methylated SCD2 with methylated SFRP1 and/or SFRP2, with overall CRC sensitivities of 88.6–91.5% and specificities of 86.1–97.3%, alongside APL sensitivities of 60.0–89.2% [41, 58, 69, 79]. The best performing panel including methylated PRIMA1, SDC2, SFRP1, and SFRP2, was reported by Bartak et al. [41]. This plasma-based 4-biomarker panel achieved 91.5% (43/47) and 97.3% (36/37) overall CRC sensitivity and specificity, alongside the relatively high APL sensitivity of 89.2% (33/37).

#### mSDC2 and mTFPI2 panels (3 studies)

Three biomarker panels including methylated SCDC2 alongside methylated TFPI2 achieved remarkable performances in terms of their overall CRC sensitivities and specificities which ranged from 90.0 to 94.9% and 94.0–98.1% respectively. The APL sensitivities showed promising performance ranging from 70.0–100% [62, 73, 77]. The ColoCaller, a recent stool-based 4-biomarker panel of methylated NDRG4, SDC2, TFPI2, and WIF1, showed encouraging performance for overall CRC detection with 94.9% (37/39) sensitivity and 98.1% (105/107) specificity. Stage-based detection (I–IV) was 88.9%, 93.8%, 100%, and 100%, accordingly. The APL performance was perfect with 100% (6/6) detection rate, the highest accuracy of the cohort, highlighting its early screening potential [62]. Another 4-biomarker panel of KRAS, methylated NDRG4, SDC2, and TFPI2 (along with β-actin reference gene), displayed a higher performance when compared to other biomarker combinations, with CRC sensitivities and specificities of 90.0% (45/50) and 94.0% (47/50). However, compared to ColoCaller, its APL sensitivity of 70.6% (36/51) was lower [73].

#### mLASS4, mLRRC4, mPPP2R5 C, mZDHHC1, and haemoglobin panels (2 studies)

Two recent biomarker panels incorporating methylated LASS4, LRRC4, PPP2R5 C, ZDHHC1, and Haemoglobin demonstrated strong overall CRC detection, with sensitivities of 93.0–95.2% and specificities of 88.5–89.8% [49, 51]. APL detection was moderate, ranging from 48.4–57.2%. The Gagrat et al. panel [49] performed best, achieving 95.2% (-/112) CRC sensitivity and 89.8% (-/176) specificity, with CRC stage-based detection (I–IV) of 93.9%, 94.7%, 100%, and 100%. Its 57.2% (-/98) APL sensitivity was higher than the 48.4% (282/583) observed in Imperiale et al. [51], though both were suboptimal compared to other biomarker combinations.

#### Other panels (5 studies)

A 5-biomarker panel, consisting of the mutational targets APC, Bat-26, KRAS, L-DNA, and p53 by Ahlquist et al., achieved a high performance for overall CRC detection with 91.0% (20/22) sensitivity and 93.0% (26/28) specificity, with an APL detection rate of 82.0% (9/11) [38]. Multi-gene panels of 20 + biomarkers was assessed by 2 studies. Ciaroloni et al. used a panel with 29 genes, CEA and CYFRA21-1 and Li et al. an 80-marker panel including methylated SEPT9 and IKZF1, achieving a similar performance for CRC detection with sensitivities of 78.1% and 77.0%, and specificities of 92.2% and 90.0%, respectively. Despite the specificities above 90%, sensitivities are inferior compared to other potentially more cost-effective candidate panels with less biomarkers [47, 56]. Furthermore, a 4-biomarker panel was the only one combining methylated BMP3, NDRG4, SDC2 with FOBT (and not FIT), achieving a moderately good performance for detection of CRC, 85.4% sensitivity and 92% specificity, and all adenomas (85.7% sensitivity). Unfortunately, as with several other studies, the reporting style could not distinguish specific detection rates for advanced precancerous lesions, but only for all adenomas or polyps in general [71]. Lastly, a 2-biomarker panel by Zhang et al. with methylated SFRP2 and WIFI1 demonstrated the fourth highest detection rate for APL (80%, 12/15) with similar sensitivity for overall CRC detection 81.3% (39/48) [76].

## Discussion

This review systematically assessed genetic and epigenetic biomarker panels using ctDNA from blood, stool, or urine, identifying promising combinations for CRC and APL detection. While several panels displayed satisfactory performance for overall CRC detection, few excelled in both CRC and APL detection, notably those combining mSDC2 with mSFRP1/2 (CRC: 91.5%/97.3%, APL: 89.2%) or mTFPI2 (CRC: 94.9%/98.1%, APL: 100%), and a 5-biomarker panel (APC, Bat-26, KRAS, L-DNA, p53; CRC: 91.0%/93.0%, APL: 82.0%). Panels such as Cologuard (including KRAS, mBMP3, mNDRG4, FIT) and mSEPT9 combinations had suboptimal APL sensitivities (up to 57% and 47.8, respectively).

The heterogeneous nature of CRC, involving various genetic and epigenetic alterations, necessitates the exploration of combinatory biomarker panels for rapid detection of malignant neoplasms and advanced precancerous lesions using liquid biopsies. High-sensitivity and high-specificity screening methods are crucial due to the morbidity and mortality associated with late CRC diagnosis, as well as the low uptake and drawbacks of available invasive or semi-invasive methods [20, 21]. Additionally, the limited potential of currently approved biomarker panels for detecting advanced precancerous lesions, underscores the need to summarise the performance of kits in the literature [31, 49, 51, 65].

The highest overall performing panels in terms of both CRC and APL detection consisted of methylated SDC2 in combination with methylated SFRP1/2 or TFPI2. A plasma-based 4-biomarker panel by Bartak et al. [41] including methylated PRIMA1, SDC2, SFRP1, and SFRP2, achieved an excellent overall CRC sensitivity and specificity of 91.5% (43/47) and 97.3% (36/37), along with a promising APL sensitivity of 89.2% (33/37). In contrast, combination of methylated SCD2 with SEPT9 in ColoDefense® panel yielded a high CRC sensitivity and specificity of 88.9% (104/117) and 92.8% (154/166), however with a moderate APL detection accuracy of 47.8% (11/23) [78]. Nevertheless, these findings clash with suggestions that combination panels with methylated SEPT9 have the highest potential for early CRC detection, as certain non-mSEPT9 combinations show superior performance for both APL and CRC detection [80]. Similarly, the panel variants of 4-biomarker stool test Cologuard (KRAS, mBMP3, mNDRG4, FIT) managed to achieve performances above 90% for overall CRC sensitivity and specificity, with suboptimal performances for APL detection, compared to other options, with the highest detection rate of 57% (65/114) by Lidgard et al. [57]. The performance data suggest that Cologuard combinations may not be the optimal diagnostic choice for APL. Similarly, FOBTs alone have even less diagnostic potential for APLs due to their poor sensitivity, but despite this their cost-effectiveness and lack of currently approved alternatives renders them usable in non-colonoscopy compliant populations for detection of advanced colorectal neoplasms. Importantly, some form of screening is preferable to none to identify cases in the early asymptomatic stage.

Another example of a high-performance panel is, ColoCaller, a recently reported stool-based 4-biomarker panel of methylated NDRG4, SDC2, TFPI2, and WIF1, exhibiting excellent performance for overall CRC (94.9% sensitivity, 37/39 and 98.1% specificity, 105/107) and APL detection (100% sensitivity, 6/6) [62]. Similarly, two other biomarker panels in the 2-biomarker combination cohort of methylated SDC2 and TFPI2 exhibited CRC detection rates of 90% and over, with 70.6% APL detection for Yang et al. [73] and 81.3% all adenomas detection (no APL-specific data) for Zhang et al. [77], emphasising the strong diagnostic capability of this biomarker combination. Lastly, a 5-mutational biomarker panel (APC, Bat-26, KRAS, L-DNA, and p53) achieved slightly improved sensitivity for APL at 82.0%, with high performance for overall CRC detection (91% sensitivity, 93% specificity), highlighting its diagnostic potential [38]. The panel consists exclusively of genetic biomarkers, and there have been no follow studies or updated panel candidates to re-assess its accuracy in the last two decades, apart from Tagore et al. [70].

Several studies have focused on ctDNA sources and their implications. Stool ctDNA has been utilised in early diagnostics, as the sample is physically more proximal to the colorectal disease compared to blood samples, with early-stage lesions developing predominantly within the mucosa and shedding into the colonic lumen. However, stool ctDNA extraction can be challenging due to low ctDNA concentrations compared to surrounding microbial DNA in stool samples [27]. Similarly, challenges arise with plasma, as it has a lower ctDNA-to-total cell-free DNA ratio, originating from clonal haematopoiesis, which is affected by age and radiotherapy, creating background noise [27, 81]. Additionally, plasma ctDNA can suffer from improper separation, prolonged storage, and repeated freeze–thaw cycles, affecting DNA integrity mainly in retrospective studies [82, 83]. Large-scale studies should aim for prospective sample collection with minimal storage prior to plasma analysis.

Apart from ctDNA concentrations and storage implications, patient preference and compliance can influence the choice of ctDNA source. Biomarker panels generally increase colorectal cancer screening compliance in previously non-compliant populations. Specifically, blood-based testing (mSEPT9) was preferred by 93.5% of participants in underserved populations, compared to 6.5% for stool-based methods (FIT) [84]. Testing rates increased from 12.6% with FIT to 93.5% with the blood test, suggesting that convenience and same-day sample collection align better with patient expectations. This strong preference for blood-based panels over stool-based ones indicates a potential strategy for improved adherence and cost-effective public health screening.

Limitations of the included studies primarily revolve around study design and methodology. Most were cross-sectional case–control studies with small sample sizes, and only a few were larger cohort studies, such as Imperiale et al. [31]. No study had a randomised design to assess the clinical benefit of their biomarker panel. According to QUADAS-2, many studies lacked detailed information on patient selection (e.g., sampling method, recruitment timing), potentially introducing bias and uncertainty about the true study design. Inconsistencies in reporting panel performance, particularly for APL, were noted, with some studies only reporting on adenomas or polyps or not at all. Additionally, methods for extracting and quantifying ctDNA varied widely with different cut-off points to balance sensitivity and specificity. There are yet no agreed standards for these methods.

While we systematically searched four major databases and performed citation searching, it is possible that some relevant ctDNA panels were not identified due to the nature of study reporting. Most studies included used a cross-sectional case–control design with relatively small sample sizes and varied in methodological reporting quality. Cost-effectiveness analysis of ctDNA panels and use of other liquid biopsy markers (e.g., cell-free nucleosomes, miRNAs, CTCs, proteomics) were beyond the scope of our review, yet remain crucial considerations from both an economic and future research perspective. Lastly, heterogeneity in panel types, analysis methods, and population characteristics precluded a meta-analysis.

## Conclusion

This review identified high-performance ctDNA biomarker panels with exceptional diagnostic accuracy for both CRC and APL, highlighting their potential for public health screening. The highest APL detection rates were observed in panels including mSDC2 with mSFRP1/2 or mTFPI2, as well as a five-biomarker mutational panel targeting APC, Bat-26, KRAS, L-DNA, and p53. Importantly, Cologuard and variant panels incorporating KRAS, mBMP3, mNDRG4, FIT, and combinations including mSEPT9, exhibited suboptimal APL sensitivity. Future research should prioritise large-scale, cross-sectional cohort or randomised studies to validate proposed ctDNA panels for clinical use. Additionally, improved study reporting is essential, including detailed methodologies with chronological information and consistent data presentation, as illustrated in our tables, to enhance research quality and facilitate data aggregation for future reviews and meta-analyses.

## Data Availability

Data supporting the study are available from the corresponding author on reasonable request via e-mail.
